# Metabolomics of blood reveals age-dependent pathways in Parkinson’s Disease

**DOI:** 10.1186/s13578-022-00831-5

**Published:** 2022-07-06

**Authors:** Nicola D’Ascenzo, Emanuele Antonecchia, Antonella Angiolillo, Victor Bender, Marco Camerlenghi, Qingguo Xie, Alfonso Di Costanzo

**Affiliations:** 1 Huazhong University of Science and Technology, Wuhan, China; 2 Department of Medical Physics and Engineering, Istituto Neurologico Mediterraneo NEUROMED I.R.C.C.S., Pozzilli, Italy; 3 Centre for Research and Training in Medicine of Aging, Department of Medicine and Health Sciences “V. Tiberio”, University of Molise, Campobasso, Italy; 4 NIM Competence Center for Digital Healthcare GmbH, Berlin, Germany; 5 University of Science and Technology of China, Hefei, China

**Keywords:** Parkinson’s Disease, Metabolomics, Age, Biomarkers

## Abstract

**Background:**

Parkinson’s Disease (PD) is the second most frequent degenerative disorder, the risk of which increases with age. A preclinical PD diagnostic test does not exist. We identify PD blood metabolites and metabolic pathways significantly correlated with age to develop personalized age-dependent PD blood biomarkers.

**Results:**

We found 33 metabolites producing a receiver operating characteristic (ROC) area under the curve (AUC) value of 97%. PCA revealed that they belong to three pathways with distinct age-dependent behavior: glycine, threonine and serine metabolism correlates with age only in PD patients; unsaturated fatty acids biosynthesis correlates with age only in a healthy control group; and, finally, tryptophan metabolism characterizes PD but does not correlate with age.

**Conclusions:**

The targeted analysis of the blood metabolome proposed in this paper allowed to find specific age-related metabolites and metabolic pathways. The model offers a promising set of blood biomarkers for a personalized age-dependent approach to the early PD diagnosis.

**Supplementary Information:**

The online version contains supplementary material available at 10.1186/s13578-022-00831-5.

## Background

Parkinson’s disease (PD) is the second most frequent neurodegenerative disorder. The Global Burden of Disease Study estimates the prevalence of PD to be approximately 6.2 million people worldwide, with a prediction of nearly 13 million by 2040 [1]. Although the connection between the accumulation of α-synuclein and the PD pathology has been extensively studied [2], the multiple mechanisms contributing to its pathogenesis, including protein misfolding and aggregation, mitochondrial injury, oxidative stress and inflammation, are not fully understood yet. As a result, a PD diagnosis is only possible after the insurgence of mobility functions impairment, such as the well-known tremor, following a consistent loss of dopaminergic neurons in the substantia nigra and in the striatum. However, at sub-clinical stage, PD is accompanied by a non-specific symptomatology including depression, sleep disturbance, or loss of olfactive sense, which most often remain unaddressed, due to the absence of a sub-clinical diagnostic PD test [3, 4].

The understanding of the relationship between PD and age has the potential to play a significant role in the personalized early PD diagnosis [5]. Sub-clinical PD manifestation occurs early, up to 20 years before the first clinical symptoms, with a risk increasing with age. This suggests that a series of age-related disfunctions may contribute to the development of PD. Proteins accumulation, genetic factors, autophagy, mitophagy, and lower protection against oxidative stress have been proposed as possible age-related PD factors [6–9].

Against this background, the blood metabolome contains a rich source of information. In fact, the metabolic pathways affected by PD originate metabolites, which are released in the blood stream. The concentration of a series of amino acids, fatty acids, acylcarnitine, lipids, purines, organic acids, and sugars revealed PD-related disfunctions of the metabolism of branched chain amino acids, tryptophan, lipid, energy, and purine, and of oxidative stress/redox homeostasis metabolic pathway [10]. Blood metabolome reflects in-vivo physiological states influenced by genetics, epigenetics and lifestyle and is therefore intimately connected with the biological age. It has been found that antioxidants, nitrogen and muscle- or kidney-related elements indicate characteristic age-related accumulation and deficiencies of metabolites [11]. Although PD is an age-related neurodegenerative disorder [12], an evidence of age-related PD significant metabolite disorder has not been found until now. Besides blood, metabolites contained in saliva, cerebrospinal fluid (CSF) and sebum have been investigated. Although a series of metabolic pathways such as carnitine shuttle, valine, leucine and isoleucine degradation, fatty acid biosynthesis, sphingolipid, arachidonic acid, primary bile acids, fatty acids, ether lipids and vitamin E metabolism have been identified as possible metabolic pathways deeply related with PD, no significant correlation between the involved metabolites, PD diagnosis and age was observed [10, 13–16].

The main aim of this paper was to study a possible relationship between PD and age by identifying blood metabolites, which express a significant age-dependent pattern in PD. This would allow to develop personalized age-dependent biomarkers. We used a supervised machine learning approach to find novel blood PD biomarkers by comparing the blood metabolome of PD and healthy subjects. We further applied a statistical analysis based on unsupervised machine learning and Bonferroni correction to discover possible age correlations of the selected biomarkers. We finally applied an enrichment pathway analysis to the selected biomarkers to identify the metabolic pathways related to age, either in PD or in healthy patients.

## Methods

### Sample participants

The subjects included in this study were consecutively enrolled at the Centre for Research and Training in Medicine of Aging of the University of Molise. We recruited 39 patients affected by PD and 39 healthy controls (HC). The subjects followed the same Mediterranean alimentation, without any specific dietary requirement. The demographic and clinical characteristics of the two groups are described in Table 1. PD patients were included in the study if the following conditions were verified:

1. A “clinically established” diagnosis of PD according to the criteria published by the Movement Disorder Society (MDS) [17]
2. Mini Mental State Examination (MMSE) score higher than or equal to 24;
3. Clinical Dementia Rating (CDR) scale score lower than or equal to 6;
4. Treatment with L-DOPA for at least 3 months.

**Table 1:** Description of the participants included in the statistical analysis

Parameter	HC	PD
Age (years)	73 ± 7.1	71 ± 6.4
Gender (Male/Female)	27/12	27/12
Scholarity	12.1 ± 3.9	11.02 ± 4.1
MMSE	28.5 ± 2.4	25.4 ± 2.3
UPDRS		55.3 ± 24.4
Hoehn and Yahr score		2.4 ± 0.6
GDS	2.5 ± 2.4	4.7 ± 3.0
BMI (Kg/m^2^)	26.3 ± 2.0	26.5 ± 2.3
Smoke	62%	30%
Alcohol	50%	44%
Hypertension	41%	42%
Diabetes	13%	15%
Dysplidemia	45%	32%
TIA/stroke	3%	12%
Myocardial infarction	5%	9%
Antihypertensive drugs	42%	46%
Hypoglycemic drugs	13%	11%
Lipid-lowering drugs	44%	22%
Antiplatelet drugs	11%	22%

We assessed the degree of PD severity with the MDS-revised Unified Parkinson's Disease Rating Scale (MDS-UPDRS) and the Hoehn and Yahr scale [18]. The study was conducted in accordance with the ethical principles stated in the Declaration of Helsinki, and with approved national and international guidelines for human research. The Institutional Review Board (IRB) of the University of Molise reviewed and approved the study (IRB Prot. n. 17/2020). A written informed consent was obtained from each participant.

### Sample collection

The blood sampling was performed between 8:00 and 8:30 a.m. after an overnight fasting of at least 8–10 h. Antecubital venous blood was collected in vacutainer tubes for plasma preparation (Becton & Dickinson, Milan, Italy) and immediately centrifuged to obtain plasma samples, which were stored at -80 °C until shipment to the analytical laboratory of BIOCRATES Life Sciences AG. The metabolomic approach based on mass spectrometry was used to obtain a quantitative determination of 630 endogenous metabolites, grouped in different biochemical classes for each plasma sample (MxP^®^ Quant 500 kit). All pre-analytical and analytical procedures related to this project were performed, documented, and reviewed according to BIOCRATES Life Sciences AG’s ISO 9001: 2015 certified quality management guidelines and standards. We used the MxP^®^ Quant 500 kit (Biocrates) for the quantification of several endogenous metabolites of various biochemical classes. Lipids and hexoses were measured by flow injection tandem mass spectrometry (FIA-MS/MS) analysis using a QTRAP^®^ 5500 instrument (AB Sciex, Darmstadt, Germany) with an electrospray ionization (ESI) source. Other metabolites were measured by liquid chromatography coupled to tandem mass spectrometry (LC–MS/MS) also using the QTRAP^®^ 5500 Instrument. A summary of the 630 metabolites measured for this study is reported in the Additional file 1: Table S1. We removed dyhydroxy-phenilalanine and tyrosine from the list of metabolites as they were clearly related to the specific therapy of PD patients.

### Statistical analysis

We pre-processed the metabolomic dataset, by reducing each variable to zero average and unitary variance. We compared two approaches for the assessment of the variation of the metabolome between the two groups of subjects. Partial least squares-discriminant analysis (PLS-DA) is a regression technique for modeling the relationship between the metabolomic data block and the respective labeled categories (PD = + 1; HC = − 1) by maximizing their covariance [19]. The PLS algorithm reduces the input metabolomic data to n_pls_ independent components and a regression coefficient matrix is used to predict the labels [20]. We compared the PLS-DA model with the orthogonal projections to latent structures (OPLS) approach [21], which provides a higher level of sophistication. It separates in fact the metabolomic dataset in two different parts. The first one consists of n_opls_ predictive components, which are correlated with the expected categories. The second one consists of a n_ort_-dimensional orthogonal non-predictive block, which is generated by the intrinsic variability of the samples and is not correlated with the expected categories. We estimated the number of optimal components by performing permutation tests for regression metrics and two-tailed permutation tests for each metabolite relative to its loading. We calculated the P-value of the PLS-DA and OPLS models by using a resampling with replacement (bootstrapping) validation technique. It is known that the higher is the number of components used to model the metabolomic dataset, the higher will be the probability of overfitting. We adopted a K-fold cross-validation method by comparing the goodness of fit R^2^Y and the predictability Q^2^ parameters after a random variation of the labels [22]. If the R^2^Y parameter after permutations is systematically higher than in the case of the correct data, then the model exhibits an illogic overfitting ability to predict any random permutation of the labels. Finally, we selected the best predictive model and the number of components avoiding overfitting and we calculated for each metabolomic parameter the variable influence on projection (VIP). However, the use of VIP values in the context of the selection of significant parameters needs a dedicated validation. There are in fact two problems. The first one is related to the intrinsic statistical fluctuations of the VIP value itself. Although the R^2^Y and Q^2^ parameters are calculated above by random label permutation and bootstrapping, that is not enough to prevent random fluctuations of the VIPs due to the highly variable nature of the data. There is therefore a certain probability that repeating the same test with an independent dataset may provide significantly different VIP values. This is particularly true in OPLS models, which, as mentioned above, aim at separating the systematic variation contained in the data into two parts—a predictive part that is correlated to the labels and an orthogonal part that is uncorrelated to the labels. A second problem, related to the first one, is the number of subjects in the study, which reflects to the statistical significance of the findings. To address this issue, we followed a four-steps procedure:

1. *Model validation* we randomly selected 80% of the entire dataset to compose a *training sample*. As mentioned above, we performed a permutation test for regression metrics, and a two-tailed permutation test for each variable to its loading (L). VIP values are selected with P < 0.05 and |L|> 0.04. We performed the receiver operating characteristics (ROC) analysis based on the selected parameters and we estimated the 95% confidence interval of the area under the curve (AUC) by using a resample with replacing (bootstrapping) approach.
2. *VIP values validation* we calculated the VIP values of the selected parameters repeating the model training 1000 times. We used a resample with replacing (bootstrapping) approach by randomly sampling 80% of the entire dataset at each evaluation. We selected only these parameters which exhibit a VIP > 1.0 in more than 95% of the trials. This test allows to remove parameters which are mostly affected by statistical fluctuations.
3. *VIP values reduction* following [23], we further strengthened the selection of the significant parameters by applying a features reduction strategy based on the principal components analysis (PCA). We analyzed the group of parameters selected at step (2) and we identified the number N_pca_ of independent principal components f_i_ accounting for 80% of the total variance. We extracted the metabolites with the most important contribution to each f_i_, identified as the ones with PCA coefficient $\left|{c}_{j}\right|>0.9\mathrm{max}\left|{c}_{i}\right|$.
4. *Metabolites selection* We finally selected as significant only the metabolites which have both a validated VIP in step (2) and a significant PCA coefficient in step (3).

### Age dependence

We calculated the correlation between the principal components f_i_ and the age of the patients by using a Spearman correlation statistical method. We selected only those significant principal components exhibiting a correlation coefficient |r|> 0.5 (P < 0.05/N_pca_). We applied a Bonferroni correction taking into account the multiple comparisons between independent statistical tests [24–29]. We finally verified that the metabolites selected in (4) also exhibit an age correlation in correspondence to the principal component in which they have a significant contribution. Data and software have been included in RADIOLYTX (www.radiolytx.com) and in the HPE Ezmeral platform (www.hpe.com/us/en/software.html).

### Pathway analysis

We performed an enrichment analysis with the software MetaboAnalyst (Version 4.0). Significant features were mapped into the Kyoto Enciclopedia of Genes and Genomes (KEGG). Feature hits on known metabolite networks were tested against a null distribution produced from permutations to yield significance values of metabolites enriched within any given network [30]. We performed first the enrichment analysis on the entire set of selected and validated metabolites with VIP > 1.0. At a second step, we restricted the analysis to these principal components f_i_ exhibiting a significant age dependence. We extracted the metabolites with the most important contribution to each f_i_, identified as the ones with PCA coefficient $\left|{c}_{j}\right|>0.9\mathrm{max}\left|{c}_{i}\right|$. We applied the enrichment analysis to each group of significant metabolites characterizing each component f_i_ and we identified possible age-dependent metabolic pathways for PD.

## Results

### Analysis of patient metadata

An overview of the clinical and demographic data of the patients is reported in Table 1. Two tailed t-test did not reveal any significant difference in age and BMI between PD patients and HC (P = 0.054 and P = 0.194, respectively). The Mini Mental State Examination (MMSE) score was found slightly significantly lower in PD group (P = 0.047). The ratio between men and women was 2:1 in the PD group, confirming the male prevalence of the disease reported in previous studies [31]. We selected a HC group with the same composition to avoid bias. Other indicators, such as smoke, scholarity, alcohol consumption, hypertension, diabetes, and dyslipidemia are not found significantly related to PD.

### Data driven prediction of PD

**Fig. 1:**
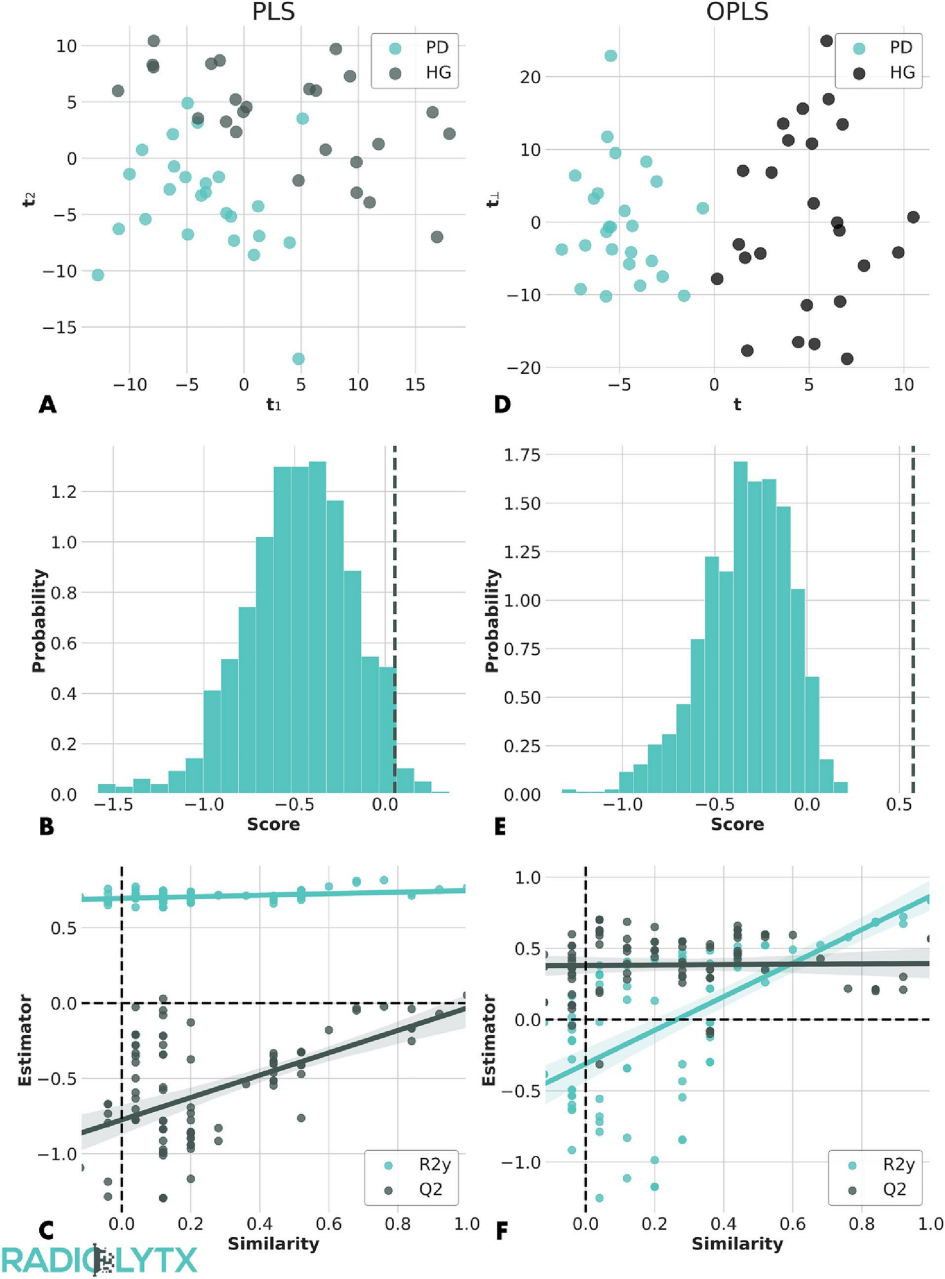
Data driven modeling for the discrimination between PD and HC subjects. The PLS model exhibits a good separation between the two categories of subjects (**A**) and its goodness is apparently confirmed by a resampling with replacement validation technique (**B**). However, we observe a non-negligible overfitting (**C**). With respect to PLS, the OPLS model exhibits a better separation between the two classes (**D**), confirmed with a P-value of 9 × 10^–4^ (**E**) and absence of overfitting (**F**)

We reported the scores plot of the first two components of a PLS-DA model with n_pls_ = 3 in Fig. 1A. The PD patients and HC subjects appeared well separated. The model had an acceptable R^2^Y = 0.76, but a very poor Q^2^ = 0.06. A resampling with validation replacement technique (n = 500) confirmed that the model described the metabolomic data with a P-value of 0.02 (Fig. 1B). The reason of such a poor predictability was due to a sizable overfitting. As shown in Fig. 1C, a random permutation of the label vectors did not affect the goodness of the fit and the R^2^Y value. We verified that lowering the number of components did not improve the performances of the PLS-DA technique.

The OPLS model with 3 orthogonal and 1 predictive component exhibited a better performance than PLS-DA, as qualitatively visible in the scores plot (Fig. 1D). Such an excellent disentanglement between PD and HC subjects was confirmed by an R^2^Y = 0.83 and a Q^2^ = 0.57. The model was validated with p-value of 9 × 10^–4^ (Fig. 1E). Finally, the goodness of fit and the R^2^Y values were significantly affected by random permutations of the labels, therefore confirming the absence of overfitting (Fig. 1F). In particular, the smaller was the similarity between the randomly permuted and the original labels, the smaller was the R^2^Y. We verified that increasing the number of orthogonal or predictive components would increase the probability of overfitting. We therefore concluded that OPLS with 3 orthogonal and 1 predictive component was the best suited model for the metabolomic data considered in this study.

### Selection of significant metabolites

**Fig. 2:**
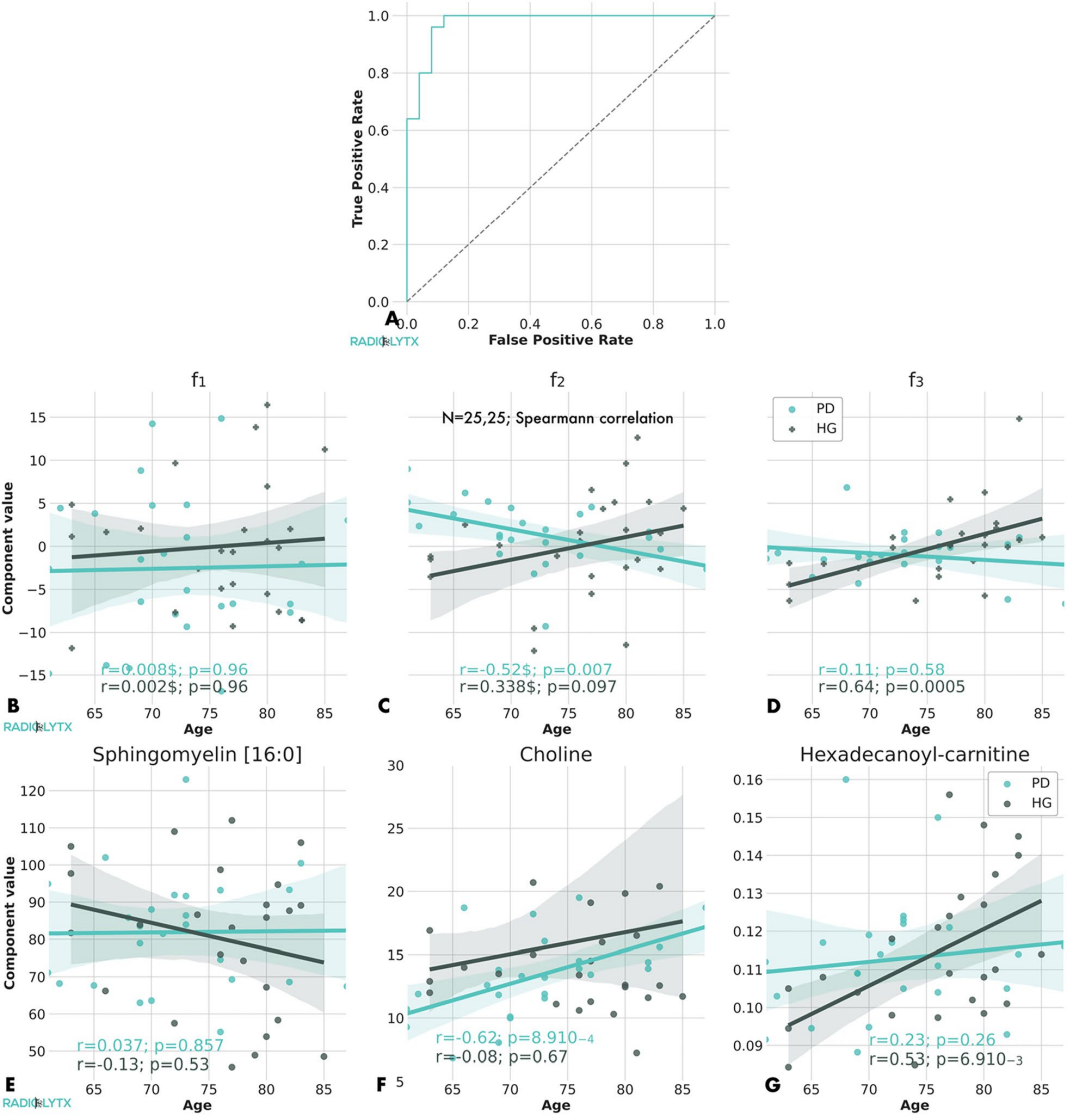
The OPLS model with 3 orthogonal and 1 predictive components can distinguish between PD and HC with an AUC in the range (0.97, 1.0) at a 95% confidence level (**A**). The principal component analysis applied to the significant metabolic parameters with VIP > 1 identifies 5 components expressing up to 80% of the total variance. In particular, f_0,1,4_ does not exhibit any age dependence (**B**), f_2_ exhibits a age correlation only for PD patients (**C**) and f_3_ exhibits a age correlation only for HC subjects (**D**). Sphingomyelin (**E**), Choline (**F**) and Hexadecanoyl-carnitine (**G**) are among the main contributions to the three principal components

The four steps of the parameter selection are shown in Table 2 and in the Additional file 1: Table S2. At a first step, we used the variable importance in projection (VIP) parameter to select those metabolomic parameters playing a significant role in the discrimination between PD and HC group. A total of 320 metabolites exhibited VIP > 1. We performed a receiver operating characteristics (ROC) analysis of this model restricted to these significant parameters (Fig. 2A). The 95% confidence interval of the area under the curve (AUC) was (0.972, 1.0), confirming the excellent discrimination power of the OPLS predictive model.

**Table 2:** Selection of the significant metabolites

Type	Name	f_i_	C	Name	f_i_	C	Name	f_i_	C
Acylcarnitines	Carnitine		0.9	Tiglyl-		0.0	**Hexadecanoyl**	**3**	0.9*
	**Acetyl-**	**3**	0.9*	Pimelyl-		1.1*	**Hexadecenoyl**	**3**	0.8*
	Butenyl-		1.2*	Dodecenoyl-		0.5*	**Octadecenoyl**	**3**	0.8*
	Methylmalonyl		2.4	**Tetradecenoyl**	**1**	0.8*			
Alkaloids	Trigonellyne		1.1						
Amine oxids	Trimethylamine N-oxide		1.7*						
Amino acid related	Asymmetric Dimethylargin		0.9	Citrulline		1.1	Kynurenine		0.8
	**Methionine-sulfoxide**	**2**	0.8*	1-Methylhistidine		0.8			
Amino acids	Cysteine		1.2	Glutamine		1.1	Glutamic Acid	2	0.8
	Threonine		1.2*						
Bile acids	Cholic acid		2.0	Chenodeoxy acid		1.9*	Deoxycholic acid		1.4
	Glycolithochol acid*		1.4	Glycolithochol Acid sulfate		1.2*			
Biogenic Amines	Gamma-amino butyracid		0.8*	Putrescine		1.6*	Serotonin		0.2
Ceramides	d16:1/24:0		1.3	d18:1/20:0	1	1.2	d18:1/26:1		1.3*
	d18:1/16:0	1	1.1	d18:1/24:0		1.2	d18:2/18:0	1	1.1
	d18:1/18:0	1	1.2	**d18:1/24:1**	**1**	1.3*	d18:2/20:0		1.2*
	d18:0/26:1OH		10.3	d18:1/24:4		1.2	d18:2/24:0	1	1.2
	d18:1/20:0 (OH)		1.2	d18:1/22:0	1	1.2	d18:2/24:1	1	1.2
Cholesteryl esters	16:1		0.9	22:5		1.1*	20:4	1	0.9
	17:1		1.1	**20:0**	**2**	0.7*	**22:1**	**2**	0.8*
	**18:1**	**1**	0.9*	**20:1**	**2**	0.7*	**22:2**	**2**	0.7*
Cresols	p-Cresol sulfate		1.2						
Diglycerides	16:1_18:0		0.8	16:1_18:1		0.9	18:2_18:4		0.7
	16:0_18:1		0.9	17:0_18:1		0.9	18:1_18:1	2	0.9
	16:0_20:3		0.8*	16:1_20:0		4.6*	18:2_18:2		1.4*
Dihexosyl ceramides	d18:1/14:0		1.1	d18:1/20:0	1	1.2	d18:0/26:1		1.2
	**d18:1/16:0**	**1**	1.2*	d18:1/24:0	1	1.1			
	d18:1/18:0	1	1.2	**d18:1/24:1**	**1**	1.1*			
Fatty acids	**Arachnidonic**	**2**	0.6*	**Docosahex**	**3**	0.7*	Eicosapent		0.7*
	Eicosatrienoic		0.3*	**Myristic**	**3**	0.2*	Eicosenoic		0.6*
	**Octadecadienoate**	**3**	0.8*						
Hexosyl ceramides	**d18:1/24:1**	**1**	1.2*	d18:1/23:0	1	1.1	d18:2/18:0	1	1.2
	d18:1/18:1	1	1.1	d18:1/26:0	1	1.1	d18:2/23:0	1	1.2
Hormones	Cortisol		1.2*	Cortisone		0.9			
Indoles	Indoleacetic acid		1.7*	Indolepropionic acid		0.7	Indoxylsulfate		0.7*
Lyso-phosphatidyl	**LysoPC(17:0)**	**2**	1.1*	LysoPC(18:2)		1.2*	LysoPC(28:1)		0.8*
Phosphatidyl-cholines	aaC34:1	1	1.1	aaC36:3	1		aeC32:2	1	1.1
	aaC32:0	1	1.1	aaC38:1	1	1.1	aeC34:3	1	1.2
	aaC32:2	1	1.2	**aaC42:0**	**1**	1,2*	aeC34:2	1	1.2
	aaC34:2	2	1.2	**aeC30:2**	**4**	0.8*	aeC44:6	2	1.2
	aaC34:3	2	1.1	**aaC42:1**	**1**	1.2*	aeC36:3	1	1.3
	aaC36:2	1	1.1	aeC38:4	1	0.9	aeC38:0	4	1.1
	aeC44:4	2	1.2	**aeC40:5**	**2**	1.1*	**aeC40:2**	**2**	1.2*
	**aeC42:4**	**1**	1.1*						
Sphingomyelins	Hydro-SM(14:1)		0.9	**SM(16:0)**	**1**	1.0*	SM(22:3)		0.6
	Hydro-SM(16:1)	1	0.9	SM(16:1)	1	0.9	SM(24:0)		0.9
	Hydro-SM(22:1)	1	0.9	SM(18:0)	1	0.9	**SM(24:1)**	**1**	0.9*
	Hydro-SM(22:2)	1	0.9	SM(18:1)	1	0.9	SM(26:0)		0.9
	Hydro-SM(24:1)	1	0.9	SM(20:2)	1	0.9	**SM(26:1)**	**1**	0.9*
Triglycerides	**(16:0_35:3)**	**0**	1.2*	**(18:2_31:0)**	**0**	1.2*	**(18:2_35:1)**	**0**	1.3*
Trihexosyl ceramides	(d18:1/16:0)		1.1*	(d18:1/18:0)		1.1*	(d18:1/24:1)		1.1*
	(d18:1/26:1)		1.1*						
Vitamins	**Choline**	**2**	0.8*						

The table contains the association of each element to the respective principal component f_1_, f_2_, or f_3_ (if significant). C indicates the ratio between the concentration of the metabolite in PD and HC: values higher or lower than 1.0 indicates, respectively, elevated or decreased metabolite level in PD. The asterisk (*) indicates parameters validated with  OPLS. Parameters in bold are additionally cross-validated with PCA. The complete list of Triglycerides is in the Additional file 1: Table S2. We show here only these Triglycerides cross-validated with PCA

However, we found that not all the 320 parameters have a significant contribution to the model. A large fraction of these parameters exhibits VIP > 1 only because of statistical fluctuations. As mentioned above, we validated the significance of the parameters using a resample with replacing (bootstrapping) approach by randomly sampling 80% of the entire dataset at each evaluation and repeating the model training 1000 times. Only 76 metabolic parameters, highlighted in Table 2 and Additional file 1: Table S2, exhibited a VIP > 1.0 in more than 95% of the cases.

We performed the final validation of the selected parameters based on an unsupervised Principal Components Analysis (PCA) technique. We found that 80% of the total variance of the selected parameters could be described with five principal components. The significant parameters with PCA coefficient $\left|{c}_{j}\right|>0.9\mathrm{max}\left|{c}_{i}\right|$ are reported in Table 2 and in the Additional file 1: Table S2. The cross-validated parameters, exhibiting a validated VIP > 1 and a significant contribution to a PCA component, are 33 and are highlighted in Table 2 and in the Additional file 1: Table S2. They belong to the groups of acylcarnitines, amino acids, ceramides, cholesteryl esters, dihexosyl ceramides, fatty acids, hexosyl ceramides, lysophosphatidyl- and phosphatidylcholines, sphyngomielins, triglycerides, and vitamins.

### Age dependence of metabolites

The principal components identified in the study define three distinct groups in relation to age. The first group is represented by the principal components f_0,_ f_1,_ and f_4._ Figure 2B shows the scatter plot of the value of f_1_ versus the age for PD and HC: no significant correlation was observed (r = 0.08, P = 0.96; r = 0.02, P = 0.98) for f_1_. A similar situation was encountered for f_0_ and f_4_. The second group, more interestingly, as shown in Fig. 2C, was represented by the component f_2_ accounting for 21% of the total variance. It exhibited a clear correlation with the age of PD patients (r = − 0.52; P = 0.007) but not of HC (r = 0.33; P = 0.09). The third group, finally, as shown in Fig. 2D, was represented by the component f_3_ and accounted for the 11% of the total variance. It exhibited a clear correlation with the age of HC subjects (r = 0.64; P = 5.6 × 10^–4^) but not of PD patients (r = 0.11; P = 0.56). The main parameters contributing to the linear expansion of the principal components are reported in Table 2. They were identified as the ones with coefficients $\left|{c}_{j}\right|>0.9\mathrm{max}\left|{c}_{i}\right|$. For instance, sphingomyelin C16:0 (Fig. 2E), choline (Fig. 2F), and hexadecanoyl-carnitine (Fig. 2G) were significant examples of the three categories of metabolites.

### Pathway analysis and age in PD

**Fig. 3:**
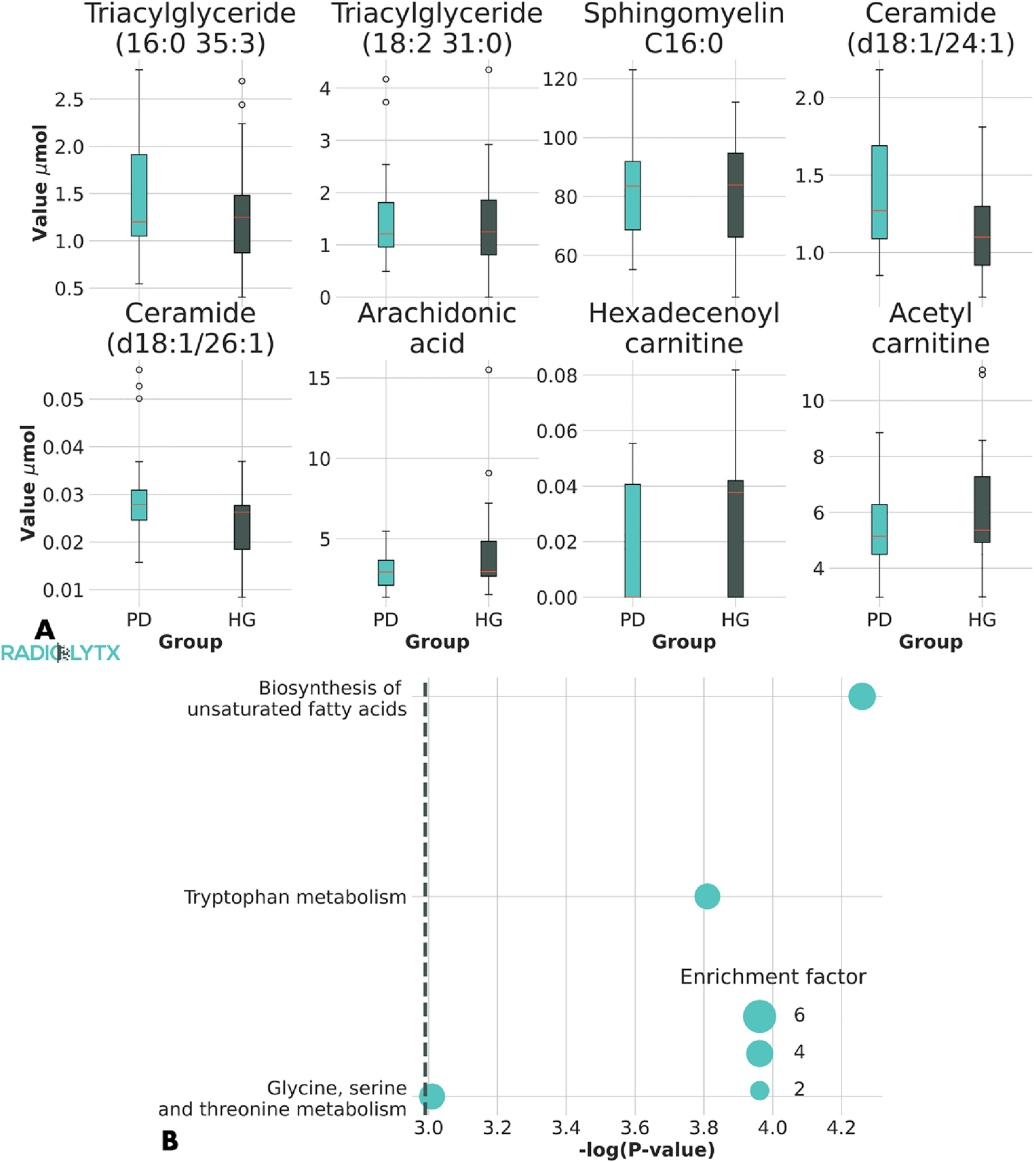
Pathway analysis. The box plots of 8 significant metabolomic parameters validated in OPLS reveal a difference of the concentration between PD and HC subjects (**A**). An enrichment analysis verifies that the selected parameters can be associated with the biosynthesis of unsaturated fatty acids, the tryptophan metabolism and the glycine, serine and threonine metabolism (**B**). We verified that the metabolites composing the first and the third pathway are correlated with age only in HG and PD subjects, respectively

We performed pathway enrichment analysis to identify the effect of age in the metabolic pathways associated to PD. At a first step we performed the analysis to the entire set of identified significant metabolites, thus neglecting any age dependence. To provide an impression of the variability of the validated parameters used at this first step of the enrichment analysis, we report the box plot of their concentration in Fig. 3A. We notice that, with respect to HC subjects, PD patients have an increased level of ceramides (d18:1/24:1) and (d18:1/26:1), triacylglycerides (18:1/32:3) and (18:2/32:1), phosphatidyl-choline aa C34:2, and putrescine. sphingomyelin C22:3 followed an opposite trend, being reduced in PD patients. As reported in Fig. 3B, the enrichment analysis reveals that the biosynthesis of unsaturated fatty acids (P = 0.004), the tryptophan metabolism (P = 0.01) and the glycine, serine and threonine metabolism (P = 0.05) are the main metabolic pathways, which express the significant selected metabolites. To verify the age dependence of these three metabolic pathways, we selected the metabolic parameters which have a significant impact into each principal component. As visible in Table 2, the parameters can be separated into three completely disjoint groups corresponding to f_1_, f_2_ and f_3,_ which address age correlation in no subjects, only in PD and only in HC, respectively. We performed a pathway analysis to the three groups distinctly and we found that metabolites not exhibiting any age correlation are associated with tryptophan metabolism. Similarly, metabolites exhibiting age correlation only in HC subjects are associated with the biosynthesis of unsaturated fatty acids. Finally, metabolites exhibiting age correlation only in PD subjects are associated with the glycine, serine and threonine metabolism.

## Discussion

### Significant metabolites as PD biomarkers

As summarized in Table 2, most of the significant metabolomic parameters presented different concentration levels in PD and HC subjects, following the expectations of the current knowledge regarding the metabolic changes in PD. For instance, a decreased level of acyl-carnitines has been found in possible association to primary decrement of mitochondrial β-oxidation and has been identified as a possible biomarker for PD [32]. Similarly, increased levels of trimethylamine N-oxide have been observed in PD patients. This amine oxide can cross the blood–brain barrier promoting cellular α-synuclein aggregations, neuroinflammation, mitochondrial dysfunction, and neuronal senescence [33]*.* As for amino acids, results are more controversial. For instance, our results confirm that no difference of glutamate concentration has to be expected in PD compared to HC [34], although an higher glutamate concentration has been indeed reported in PD patients [35, 36]. More interestingly, the importance of threonine as a possible PD biomarker is confirmed also in other studies [37]. Similarly, high levels of putrescine and ceramides in the blood of PD patients have also been observed [38, 39]. Polyamines, such as putrescine, cadaverine, spermidine and spermine, are involved in many vital processes, including cell proliferation and differentiation, gene transcription and translation, modulation of ion channels and receptors, and can promote the aggregation and fibrillation of α-synuclein [38]. Ceramides play an essential role not only in shaping cell membranes, but also in regulating cellular processes of vital importance in PD. This leads to the observation, supported and confirmed also in other studies [39], of elevated Ceramide levels in PD patients.

In general, the finding that cholesteryl esters, sphingomielins, fatty acids, dihexosylceramides, hexosylceramides, ceramides, phosphatidylcholines, lysophosphatidylcholines, diglycerides and triglycerides have a high impact in the discrimination between PD and HC indicates a general alteration of lipid metabolism in PD [40, 41], supporting the hypothesis of PD as a lipidopathy [42].

Finally, the increase of the tryglicerides levels observed in this study disagrees with the literature, which shows a reduction or no differences in PD patients compared to HC [40]. However, our result is in line with a post-mortem lipidomic study showing an increase of tryglicerides in the CSF of PD patients [43]. The ethnical and demographical characteristics of the subjects might be a possible explanation for the observed discrepancy [41].

### Glycine, threonine, and serine metabolism correlates with age only in PD patients

A striking result of our study is that metabolites related to the glycine, threonine and serine metabolism exhibit a correlation with age in PD patients, but not in healthy subjects. It is well known that, in the production process of ATP from ADP, the creatine generated from threonine provides the necessary phosphate groups. As for PD, the relationship between glycine, serine and threonine metabolism has been intensively proved at the onset of α-synuclein aggregation, when glycine, serine and threonine metabolism appear to be down-regulated [44]. However, a direct observation of the age dependency of this metabolic pathway in PD has never been reported. The fact that age-dependent mechanisms may occur in PD is corroborated by the recently observed age-dependent neuromelanin production in PD [45]. Although there is no direct association between neuromelanin, threonine, glycine, and serotonin, this finding shows that age dependent mechanisms must be expected in PD. The fact that glycine, serine and threonine metabolism have a remarkable age-dependence only in PD patients suggests that this metabolic pathway might be associated with the neurodegenerative process typical of PD. Choline is the metabolic product of glycine, serine and threonine metabolism and exhibits a striking age dependence only in PD subjects, as mentioned above. Abnormal choline transport and metabolism have been implicated in several neurodegenerative disorders. It is an essential nutrient for all cells because it plays a critical role in the synthesis of the membrane phospholipid, as well as in the synthesis of the neurotransmitter acetylcholine. Its deficiency affects the expression of genes involved in cell proliferation, differentiation, and apoptosis, and it has been associated with liver dysfunction and cancer [46]. Indirect evidence of the association of glycine metabolism to age is supported by a series of independent results. By comparing young and old human fibroblasts it was found that epigenetic downregulation of the glycine-C-acetyltransferase (GCAT) and serine hydroxymethyltransferase 2 (SHMT2) genes involved in mitochondrial glycine synthesis correspond to the aging-related loss of cellular respiration. Interestingly, the phenotype of aged cells is restored back to young cells by adding glycine to the culture media [47, 48]. The role of serine synthesis in age-related diseases has been intensively studied. It has been shown that serine can directly affect lifespan through metabolic regulation [49].

Our results also open new possible exploration pathways in the relationship between PD and other chronic neurodegenerative diseases, such as Alzheimer’s Disease (AD). Glycine, serine, and threonine metabolism is in fact one of the six metabolic pathways that distinguish HC from AD patients [50]. Furthermore, metabolomic analysis has recently shown that the cognitive impairment due to post-traumatic brain injuries is associated with aberrations in glycine, serine and threonine metabolism [51]. When it comes to age dependency, preclinical studies may provide indirect evidence. A recent metabolomics analysis was performed on triple transgenic AD (3xTg-AD) 2- and 6-month-old mice. Relevant metabolites were identified following a statistical analysis like the one presented in this paper. Glycine, serine and threonine metabolism appeared as a significant metabolic pathway in the 6-month-old mice but not in the 2-month-old mice [52]. An imbalance between excitability (aspartate and glutamine) and inhibition (GABA and Glycine) may be a signature of AD. While this finding may suggest a dependence of glycine, serine and threonine metabolism on the progression of the disease, it is not in direct relationship with age.

More suggestive implications of our results in the intimate connections between AD and PD may be found with a deeper understanding of the spleen to brain connection [53]. The splenic nerve connects to the vagus nerve, which is connected to the brain stem. In a metabolomic study on AD mice models, it has been observed that impaired glycine, serine and threonine pathways were correlated with the increase in spleen size of AD mice at 6 months of age and in control mice at an age of 24 months. More interestingly, the age dependence of glycine, serine and threonine pathways does not exhibit any difference between healthy controls and AD after 24 months, therefore suggesting the absence of any age dependency at a later stage of the disease [54]. Following this observation, although our results enhance for the first time to our knowledge a clear age-dependence of the glycine, serine and threonine metabolic pathway in PD, it will be interesting to understand the strength of this effect at later stages of the disease.

### Biosynthesis of fatty acids correlates with age only in healthy subjects

As mentioned above, the metabolites identified in this study support the strict relationship between PD and lipids. Alterations in the biosynthesis of fatty acids are not only indicative of a mitochondrial disfunction, but also of possible processes of mitophagy and apoptosis implied by the development of PD [55]. It has been observed how the integration of fatty acids omega-3 have a neuroprotective action in a model of hemiparkinsonism [56]. The effect of age on the biosynthesis of unsaturated fatty acids in the HC group is clearly connected with the emerging role of lipid metabolism [57]. For instance, age correlation of lipids and fatty acids has been found in the analysis of plasma samples from a cohort of 269 individuals [58]. The observation of a different age correlation between HC and PD agrees with other findings. By way of example, aldosterone, pantothenic acid, and Nacetyl-l-methionine were associated with age in HC; however, only 1 metabolite FFA 12:0 showed association with age in PD [59]. Therefore, the absence of correlation in PD patients may indicate an already compromised metabolism and needs further investigation.

### Tryptophan metabolism characterizes PD but does not correlate with age

The role of tryptophan in PD is well-documented. Particularly kynurenine, a key intermediate in the breakdown of tryptophan and formation of nicotinamide adenine dinucleotide (NAD +) via the kynurenine pathway (KP), is involved in a variety of physiopathological processes and diseases—including cancer, autoimmune diseases, inflammatory diseases, neurologic diseases and psychiatric disorders [60]. KP metabolites, such as quilinolinic acid, cause neurotoxicity and consequently neuronal apoptosis and neurodegeneration, while others, such as the kynurenic acid, act as neuroprotectant. Furthermore, excess levels of quinolinic acid lead to the formation of metabolite assemblies that causes ∝-synuclein aggregation, with consequent neuronal toxicity and PD [61]. Metabolites associated with tryptophan metabolism also modulate inflammation, regulate energy homeostasis and control mental health [62]. PD patients show lower kynurenic acid and higher quinolinic acid levels compared to HC, especially in advanced stages of the disease [63]. When it comes to other neurodegenerative diseases, tryptophan metabolism has been found in significant relationship with both AD and Mild Cognitive Impairment (MCI) progression [64]. Contrary to previous studies exhibiting a robust association of tryptophan metabolism with aging [65], we did not observe any significant age dependency.

## Conclusion

In conclusion, the targeted analysis of the blood metabolome proposed in this paper allowed to find specific age-related metabolites and metabolic pathways. The predictive OPLS model developed in this paper has an excellent discrimination power between PD and HC and offers a promising set of blood biomarkers for a personalized age-dependent approach to the early PD diagnosis.

## Supplementary Information

**Additional file 1: Table S1.**List of the analyzed metabolites

## Data Availability

The datasets used and/or analyzed during the current study are available from the corresponding author on reasonable request. In addition, the datasets and the analysis software will be made available in the digital healthcare platform prepared by the GATEKEEPER consortium.

## Abbreviations

ADP: Adenosine diphosphate
ATP: Adenosine triphosphate
AUC: Area under the curve
CDR: Clinical dementia rating
CSF: Cerebrospinal fluid
HC: Healthy control
IRB: Institutional Review Board
KEGG: Kyoto Enciclopedia of Genes and Genomes
KP: Kynurenine pathway
MDS: Movements Disorder Society
MDS-UPDRS: MDS-revised Unified Parkinson’s Disease Rating Scale
MMSE: Mini Mental State Examination
NAD: Nicotinamide adenine dinucleotide
OPLS: Ordered projection to latent structures
PCA: Principal component analysis
PD: Parkinson’s Disease
PLS-DA: Partial least squares-discriminant analysis
ROC: Receiver operating characteristics
TCA: Tricarboxylic acid cycle
VIP: Variable influence on projection
