# Supplementary material for: Metabolomics of blood reveals age-dependent pathways in Parkinson’s Disease
Source: Cell Biosci. 2022 Jul 6;12:102. doi: 10.1186/s13578-022-00831-5 (PMC9258166; doi:10.1186/s13578-022-00831-5)
Supplement: Supplementary file 1 — Additional file 1: Table S1.List of the analyzed metabolites [file 13578_2022_831_MOESM1_ESM.docx]

**Table S1:** List of the analyzed metabolites

| **Type** | **Names** | | | | | |
| --- | --- | --- | --- | --- | --- | --- |
| Acylcarnitines | Carnitine | Butenylcarnitine | Hexanoylcarnitine (Fumarylcarnitine) | Decadienoyl-carnitine | Tetradecadienoyl-carnitine | Hydroxyhexadeca-dienoylcarnitine |
|  | Acetylcarnitine | Valerylcarnitine | Hexenoylcarnitine | Dodecanoyl-carnitine | Hydroxytetradeca-dienoylcarnitine | Octadecanoyl-carnitine |
|  | Propionylcarnitine | Glutarylcarnitine (Hydroxyhexanoylcarnitine) | Pimelylcarnitine | Dodecanedioyl-carnitine | Hexadecanoyl-carnitine | Octadecenoyl-carnitine |
|  | Malonylcarnitine (Hydroxybutyrylcarnitine) | Methylglutaryl-carnitine | Octanoylcarnitine | Dodecenoyl-carnitine | Hydroxyhexa-decanoylcarnitine | Hydroxyoctadec-enoylcarnitine |
|  | Hydroxypropionyl-carnitine | Hydroxyvaleryl-carnitine (Methylmalonylcarnitine) | Nonanoylcarnitine | Tetradecanoyl-carnitine | Hexadecenoyl-carnitine | Octadecadienoyl-carnitine |
|  | Propenylcarnitine | Tiglylcarnitine | Decanoylcarnitine | Tetradecenoyl-carnitine | Hydroxyhexadec-enoylcarnitine |  |
|  | Butyrylcarnitine | Glutaconyl-carnitine | Decenoylcarnitine | Hydroxytetradec-enoylcarnitine | Hexadecadienoyl-carnitine |  |
| Alkaloids | Trigonelline |  |  |  |  |  |
| Amine Oxides | Trimethylamine N-oxide |  |  |  |  |  |
| Amino Acid Related | 1-Methylhistidine | Asymmetric dimethylarginine | cis-4-Hydroxyproline | Dihydroxy-phenylalanine | Nitrotyrosine | Sarcosine |
|  | 3-Methylhistidine | alpha-Aminoadipic acid | Carnosine | Homoarginine | Ornithine | Symmetric dimethylarginine |
|  | 5-Aminovaleric acid | L-Anserine | Citrulline | Homocysteine | Phenylacetyl-glycine | trans-4-Hydroxyproline |
|  | alpha-Amino-butyric acid | beta-Aminobutyric acid | Creatinine | Kynurenine | Phenylalanine betaine | Taurine |
|  | Acetylornithine | Betaine | Cystine | Methionine-Sulfoxide | Proline betaine | Tryptophan betaine |
| Amino Acid | Alanine | Cysteine | Histidine | Methionine | Threonine |  |
|  | Arginine | Glutamine | Isoleucine | Phenylalanine | Tryptophan |  |
|  | Asparagine | Glutamic Acid | Leucine | Proline | Tyrosine |  |
|  | Aspartic Acid | Glycine | Lysine | Serine | Valine |  |
| Bile Acids | Cholic Acid | Glycocholic acid | Glycolithocholic acid | Taurocholic acid | Taurolithocholic acid |  |
|  | Chenodeoxycholic acid | Glycochenodeoxy-cholic acid | Glycolithocholic acid sulfate | Taurochenodeoxy-cholic acid | Tauromuricholic acids |  |
|  | Deoxycholic acid | Glycodeoxycholic acid | Glycoursodeoxy-cholic acid | Taurodeoxycholic acid |  |  |
| Biogenic Amines | beta-Alanine | gamma-Amino-butyric acid | Phenylethylamine | Serotonin | Spermine |  |
|  | Dopamine | Histamine | Putrescine | Spermidine |  |  |
| Carbohydrates and Related | Hexose |  |  |  |  |  |
| Carboxylic Acids | Aconitic acid | Tetradecanedioic acid | Lactic acid | Succinic acid |  |  |
|  | Dodecanedioic acid | Hippuric acid | Hydroxyglutaric acid |  |  |  |
| Ceramides | (d16:1/18:0) | (d18:1/14:0) | (d18:1/20:0(OH)) | (d18:1/24:1) | (d18:2/16:0) | (d18:2/23:0) |
|  | (d16:1/20:0) | (d18:1/16:0) | (d18:1/20:0) | (d18:1/25:0) | (d18:2/18:0) | (d18:2/24:0) |
|  | (d16:1/22:0) | (d18:1/18:0(OH)) | (d18:1/22:0) | (d18:1/26:0) | (d18:2/18:1) | (d18:2/24:1) |
|  | (d16:1/23:0) | (d18:1/18:0) | (d18:1/23:0) | (d18:1/26:1) | (d18:2/20:0) |  |
|  | (d16:1/24:0) | (d18:1/18:1) | (d18:1/24:0) | (d18:2/14:0) | (d18:2/22:0) |  |
| Cholesteryl Esters | 14:0 | 16:0 | 18:0 | 20:0 | 20:5 | 22:5 |
|  | 14:1 | 16:1 | 18:1 | 20:1 | 22:0 | 22:6 |
|  | 15:0 | 17:0 | 18:2 | 20:3 | 22:1 |  |
|  | 15:1 | 17:1 | 18:3 | 20:4 | 22:2 |  |
| Cresols | p-Cresol sulfate |  |  |  |  |  |
| Diacylglyceride | (14:0_14:0) | (16:0_18:1) | (16:1_20:0) | (18:1_18:4) | (18:2_18:2) | (22:1_22:2) |
|  | (14:0_18:1) | (16:0_18:2) | (17:0_17:1) | (18:1_20:0) | (18:2_18:3) | O-(14:0_18:2) |
|  | (14:0_18:2) | (16:0_20:0) | (17:0_18:1) | (18:1_20:1) | (18:2_18:4) | O-(16:0_18:1) |
|  | (14:0_20:0) | (16:0_20:3) | (18:0_20:0) | (18:1_20:2) | (18:2_20:0) | O-(16:0_20:4) |
|  | (14:1_18:1) | (16:0_20:4) | (18:0_20:4) | (18:1_20:3) | (18:2_20:4) |  |
|  | (14:1_20:2) | (16:1_18:0) | (18:1_18:1) | (18:1_20:4) | (18:3_18:3) |  |
|  | (16:0_16:0) | (16:1_18:1) | (18:1_18:2) | (18:1_22:5) | (18:3_20:2) |  |
|  | (16:0_16:1) | (16:1_18:2) | (18:1_18:3) | (18:1_22:6) | (21:0_22:6) |  |
| Dihexosylceramides | (d18:1/14:0) | (d18:1/20:0) | (d18:1/24:1) | (18:0/18:0(OH)) | (18:0/22:0) | (18:0/26:1(OH)) |
|  | (d18:1/16:0) | (d18:1/22:0) | (d18:1/26:0) | (18:0/18:0) | (18:0/24:0) | (18:0/26:1) |
|  | (d18:1/18:0) | (d18:1/24:0) | (d18:1/26:1) | (18:0/20:0) | (18:0/24:1) |  |
| Fatty Acids | Arachidonic acid | Eicosapentaenoic acid | Myristic acid | Stearic acid | Octadecadienoate | Eicosadienoic acid |
|  | Docosahexaenoic acid | Dodecanoic acid | Palmitic acid | Octadecenoic acid | Eicosenoic acid | Eicosatrienoic acid |
| Hexosylceramide | (d16:1/22:0) | (d18:1/18:0) | (d18:1/23:0) | (d18:1/26:1) | (d18:2/22:0) |  |
|  | (d16:1/24:0) | (d18:1/18:1) | (d18:1/24:0) | (d18:2/16:0) | (d18:2/23:0) |  |
|  | (d18:1/14:0) | (d18:1/20:0) | (d18:1/24:1) | (d18:2/18:0) | (d18:2/24:0) |  |
|  | (d18:1/16:0) | (d18:1/22:0) | (d18:1/26:0) | (d18:2/20:0) |  |  |
| Hormones and Related | Abscisic acid | Cortisol | Cortisone | Dehydroepiandro-sterone sulfate |  |  |
| Indoles and Derivatives | Indoleacetic acid | Indolepropionic acid | Indoxyl sulfate | Indole |  |  |
| Lysophosphatidyl-cholines | C14:0 | C17:0 | C18:2 | C24:0 | C28:0 |  |
|  | C16:0 | C18:0 | C20:3 | C26:0 | C28:1 |  |
|  | C16:1 | C18:1 | C20:4 | C26:1 |  |  |
| Nucleobases and Related | Hypoxanthine | Xanthine |  |  |  |  |
| Phosphatidyl-cholines | aa C24:0 | aa C36:0 | aa C40:1 | ae C30:1 | ae C36:5 | ae C40:6 |
|  | aa C26:0 | aa C36:1 | aa C40:2 | ae C30:2 | ae C38:0 | ae C42:0 |
|  | aa C28:1 | aa C36:2 | aa C40:3 | ae C32:1 | ae C38:1 | ae C42:1 |
|  | aa C30:0 | aa C36:3 | aa C40:4 | ae C32:2 | ae C38:2 | ae C42:2 |
|  | aa C30:2 | aa C36:4 | aa C40:5 | ae C34:0 | ae C38:3 | ae C42:3 |
|  | aa C32:0 | aa C36:5 | aa C40:6 | ae C34:1 | ae C38:4 | ae C42:4 |
|  | aa C32:1 | aa C36:6 | aa C42:0 | ae C34:2 | ae C38:5 | ae C42:5 |
|  | aa C32:2 | aa C38:0 | aa C42:1 | ae C34:3 | ae C38:6 | ae C44:3 |
|  | aa C32:3 | aa C38:1 | aa C42:2 | ae C36:0 | ae C40:1 | ae C44:4 |
|  | aa C34:1 | aa C38:3 | aa C42:4 | ae C36:1 | ae C40:2 | ae C44:5 |
|  | aa C34:2 | aa C38:4 | aa C42:5 | ae C36:2 | ae C40:3 | ae C44:6 |
|  | aa C34:3 | aa C38:5 | aa C42:6 | ae C36:3 | ae C40:4 |  |
|  | aa C34:4 | aa C38:6 | ae C30:0 | ae C36:4 | ae C40:5 |  |
| Sphingomyelins | Hydroxysphingo-myelin C14:1 | Hydroxysphingo-myelin C22:2 | Sphingomyelin C16:1 | Sphingomyelin C20:2 | Sphingomyelin C24:1 |  |
|  | Hydroxysphingo-myelin C16:1 | Hydroxysphingo-myelin C24:1 | Sphingomyelin C18:0 | Sphingomyelin C22:3 | Sphingomyelin C26:0 |  |
|  | Hydroxysphingo-myelin C22:1 | Sphingomyelin C16:0 | Sphingomyelin C18:1 | Sphingomyelin C24:0 | Sphingomyelin C26:1 |  |
| Triacylglyceride | (14:0_32:2) | (16:0_38:5) | (17:2_34:3) | (18:1_36:0) | (18:3_34:0) | (20:3_36:5) |
|  | (14:0_34:0) | (16:0_38:6) | (17:2_36:2) | (18:1_36:1) | (18:3_34:1) | (20:4_30:0) |
|  | (14:0_34:1) | (16:0_38:7) | (17:2_36:3) | (18:1_36:2) | (18:3_34:2) | (20:4_32:0) |
|  | (14:0_34:2) | (16:0_40:6) | (17:2_36:4) | (18:1_36:3) | (18:3_34:3) | (20:4_32:1) |
|  | (14:0_34:3) | (16:0_40:7) | (17:2_38:5) | (18:1_36:4) | (18:3_35:2) | (20:4_32:2) |
|  | (14:0_35:1) | (16:0_40:8) | (17:2_38:6) | (18:1_36:5) | (18:3_36:1) | (20:4_33:2) |
|  | (14:0_35:2) | (16:1_28:0) | (17:2_38:7) | (18:1_36:6) | (18:3_36:2) | (20:4_34:0) |
|  | (14:0_36:1) | (16:1_30:1) | (18:0_30:0) | (18:1_38:5) | (18:3_36:3) | (20:4_34:1) |
|  | (14:0_36:2) | (16:1_32:0) | (18:0_30:1) | (18:1_38:6) | (18:3_36:4) | (20:4_34:2) |
|  | (14:0_36:3) | (16:1_32:1) | (18:0_32:0) | (18:1_38:7) | (18:3_38:5) | (20:4_34:3) |
|  | (14:0_36:4) | (16:1_32:2) | (18:0_32:1) | (18:2_28:0) | (18:3_38:6) | (20:4_35:3) |
|  | (14:0_38:4) | (16:1_33:1) | (18:0_32:2) | (18:2_30:0) | (20:0_32:3) | (20:4_36:2) |
|  | (14:0_38:5) | (16:1_34:0) | (18:0_34:2) | (18:2_30:1) | (20:0_32:4) | (20:4_36:3) |
|  | (14:0_39:3) | (16:1_34:1) | (18:0_34:3) | (18:2_31:0) | (20:0_34:1) | (20:4_36:4) |
|  | (16:0_28:1) | (16:1_34:2) | (18:0_36:1) | (18:2_32:0) | (20:1_24:3) | (20:4_36:5) |
|  | (16:0_28:2) | (16:1_34:3) | (18:0_36:2) | (18:2_32:1) | (20:1_26:1) | (20:5_34:0) |
|  | (16:0_30:2) | (16:1_36:1) | (18:0_36:3) | (18:2_32:2) | (20:1_30:1) | (20:5_34:1) |
|  | (16:0_32:0) | (16:1_36:2) | (18:0_36:4) | (18:2_33:0) | (20:1_31:0) | (20:5_34:2) |
|  | (16:0_32:1) | (16:1_36:3) | (18:0_36:5) | (18:2_33:1) | (20:1_32:1) | (20:5_36:2) |
|  | (16:0_32:2) | (16:1_36:4) | (18:0_38:6) | (18:2_33:2) | (20:1_32:2) | (20:5_36:3) |
|  | (16:0_32:3) | (16:1_36:5) | (18:0_38:7) | (18:2_34:0) | (20:1_32:3) | (22:0_32:4) |
|  | (16:0_33:1) | (16:1_38:3) | (18:1_26:0) | (18:2_34:1) | (20:1_34:0) | (22:1_32:5) |
|  | (16:0_33:2) | (16:1_38:4) | (18:1_28:1) | (18:2_34:2) | (20:1_34:1) | (22:2_32:4) |
|  | (16:0_34:0) | (16:1_38:5) | (18:1_30:0) | (18:2_34:3) | (20:1_34:2) | (22:3_30:2) |
|  | (16:0_34:1) | (17:0_32:1) | (18:1_30:1) | (18:2_34:4) | (20:1_34:3) | (22:4_32:0) |
|  | (16:0_34:2) | (17:0_34:1) | (18:1_30:2) | (18:2_35:1) | (20:2_32:0) | (22:4_32:2) |
|  | (16:0_34:3) | (17:0_34:2) | (18:1_31:0) | (18:2_35:2) | (20:2_32:1) | (22:4_34:2) |
|  | (16:0_34:4) | (17:0_34:3) | (18:1_32:0) | (18:2_35:3) | (20:2_34:1) | (22:5_32:0) |
|  | (16:0_35:1) | (17:0_36:3) | (18:1_32:1) | (18:2_36:0) | (20:2_34:2) | (22:5_32:1) |
|  | (16:0_35:2) | (17:0_36:4) | (18:1_32:2) | (18:2_36:1) | (20:2_34:3) | (22:5_34:1) |
|  | (16:0_35:3) | (17:1_32:1) | (18:1_32:3) | (18:2_36:2) | (20:2_34:4) | (22:5_34:2) |
|  | (16:0_36:2) | (17:1_34:1) | (18:1_33:0) | (18:2_36:3) | (20:2_36:5) | (22:5_34:3) |
|  | (16:0_36:3) | (17:1_34:2) | (18:1_33:1) | (18:2_36:4) | (20:3_32:0) | (22:6_32:0) |
|  | (16:0_36:4) | (17:1_34:3) | (18:1_33:2) | (18:2_36:5) | (20:3_32:1) | (22:6_32:1) |
|  | (16:0_36:5) | (17:1_36:3) | (18:1_33:3) | (18:2_38:4) | (20:3_32:2) | (22:6_34:1) |
|  | (16:0_36:6) | (17:1_36:4) | (18:1_34:1) | (18:2_38:5) | (20:3_34:0) | (22:6_34:2) |
|  | (16:0_37:3) | (17:1_36:5) | (18:1_34:2) | (18:2_38:6) | (20:3_34:1) | (22:6_34:3) |
|  | (16:0_38:1) | (17:1_38:5) | (18:1_34:3) | (18:3_30:0) | (20:3_34:2) |  |
|  | (16:0_38:2) | (17:1_38:6) | (18:1_34:4) | (18:3_32:0) | (20:3_34:3) |  |
|  | (16:0_38:3) | (17:1_38:7) | (18:1_35:2) | (18:3_32:1) | (20:3_36:3) |  |
|  | (16:0_38:4) | (17:2_34:2) | (18:1_35:3) | (18:3_33:2) | (20:3_36:4) |  |
| Trihexosylceramides | (d18:1/16:0) | (d18:1/18:0) | (d18:1/24:1) | (d18:1/26:1) | (d18:1/20:0) | (d18:1/22:0) |
| Vitamins and Cofactors | Choline |  |  |  |  |  |

**Table S2:** Selection of significant Triacylglycerides

| Tryacylglicerides | | | | |
| --- | --- | --- | --- | --- |
| (18:2_36:4) | (16:0_36:6) | (18:0_32:2) | _(14:0_36:1)* | (20:3_34:1) |
| (18:2_36:5) | (38_36:4) | (22:5_32:0) | (18:2_33:0)* | (16:0_35:1)* |
| (20:2_34:4) | (18:0_36:5) | (18:0_36:3) | (20:3_34:0) | (17:1_36:3) |
| (18:1_26:0) | (18:0_38:7) | (20:1_32:2) | (20:1_34:2) | (18:1_33:2) |
| (18:2_28:0) | (18:2_34:2) | (16:0_38:6) | (18:2_34:1) | (18:1_34:2) |
| (18:3_36:4) | (18:2_32:2) | (16:0_40:7) | (16:0_34:0) | (16:1_34:0) |
| (16:0_28:2) | (16:1_36:4) | (20:2_34:1) | (16:0_38:4)* | (16:1_38:3) |
| (16:0_30:2) | (22:5_32:1) | (14:0_34:3) | (18:1_32:2) | (20:1_34:1) |
| (18:2_36:3) | (18:2_38:5) | (20:5_34:0) | (20:2_32:0) | (14:0_35:2) |
| (16:0_28:1) | (18:3_32:0) | (18:3_32:1) | (20:4_34:1) | (16:1_34:2) |
| (18:0_30:1) | (18:1_36:6) | (20:4_32:2) | (18:1_38:7) | (20:1_32:1) |
| (22:2_32:4) | (18:1_30:1) | (18:3_34:0)* | (16:0_35:3)** | (18:1_32:1) |
| (18:2_34:4) | (20:5_34:2) | (14:0_34:2) | (16:0_36:3)* | (17:0_34:1)* |
| (22:6_34:3) | (18:3_34:2) | (16:0_32:1)* | (20:3_32:1) | (17:1_34:1) |
| (18:1_30:2) | (22:5_34:2) | (16:0_34:4) | (22:4_32:0) | (17:1_32:1) |
| (18:3_34:3) | (16:0_32:2) | (18:2_32:1) | (16:0_34:1) | (16:1_32:1) |
| (18:0_36:2) | (22:6_32:1) | (20:3_32:2) | (16:0_38:7) | (18:1_36:0) |
| (20:0_32:4) | (18:3_38:5) | (20:1_34:0) | (20:3_32:0) | (17:0_36:3) |
| (18:0_36:4) | (18:1_32:3)*5:33 | (20:5_34:1) | (18:2_31:0)** | (16:0_35:2) |
| (18:1_36:5) | (18:2_38:4) | (18:2_36:0)* | (18:1_34:3) | (18:1_33:0) |
| (18:2_30:1) | (18:0_34:3) | (18:2_34:0) | (16:0_38:2) | (17:1_34:2) |
| (18:2_34:3) | (18:1_30:0) | (16:0_38:1) | (20:4_34:0) | (16:1_34:1) |
| (18:1_36:4) | (22:4_34:2) | (16:0_34:3) | (20:2_32:1) | (17:0_34:3) |
| (18:3_30:0) | (20:4_34:2) | (18:3_36:2) | (16:0_33:2) | (20:4_33:2) |
| (18:3_36:3) | (20:4_34:3) | (18:2_35:2)* | (16:0_33:1) | (18:1_33:1) |
| (18:2_38:6) | _(14:0_36:3)* | (20:4_35:3) | (18:1_31:0) | (18:1_36:1) |
| (20:0_32:3) | (14:0_34:0) | (22:5_34:1) | (18:2_35:1)** | (18:1_35:3) |
| (20:3_34:3) | (18:2_33:2) | (18:1_34:4) | (16:0_34:2)* | (16:0_36:2) |
| (18:1_28:1) | (20:2_34:2) | (16:0_38:5) | (18:2_33:1) | (18:1_35:2) |
| (18:2_30:0) | (18:0_32:1) | (18:0_38:6) | (17:0_32:1) | (16:1_33:1) |
| (14:0_36:4) | (18:3_36:1) | (18:3_35:2) | (20:4_36:2) | (18:1_34:1) |
| (17:0_36:4) | (18:0_34:2) | (18:3_34:1) | (16:0_37:3) | (16:1_36:1) |
| (16:0_36:5) | (16:1_36:5) | (18:2_36:1) | (18:1_32:0)* | (16:1_36:2) |
| (16:0_40:8) | (14:0_38:5) | (16:1_38:4) | (16:1_34:3) | (18:1_36:2) |
| (20:2_34:3) | (14:0_38:4) | (16:0_40:6) | (16:1_32:2) |  |
| (14:0_32:2) | (14:0_34:1) | (20:4_32:1) | (17:0_34:2) |  |
| (18:2_35:3) | (18:2_32:0) | (16:0_38:3)* | (14:0_36:2) |  |
| (16:0_32:3) | (20:3_34:2) | (18:1_38:6) | (18:1_38:5) |  |

The asterisk (*) indicates these Tryaglicerides validated with the OPLS model. The double asterisk (**) indicates these parameters cross-validated with PCA.
